# Rapid generation of novel models of RAG1 deficiency by CRISPR/Cas9-induced mutagenesis in murine zygotes

**DOI:** 10.18632/oncotarget.7341

**Published:** 2016-02-12

**Authors:** Lisa Ott de Bruin, Wei Yang, Kelly Capuder, Yu Nee Lee, Maddalena Antolini, Robin Meyers, Martin Gellert, Kiran Musunuru, John Manis, Luigi Notarangelo

**Affiliations:** ^1^ Division of Immunology, Boston Children's Hospital, Harvard Medical School, Boston, MA, USA; ^2^ Pediatric Immunology, Wilhelmina Children's Hospital, Utrecht University Medical Center, Utrecht, The Netherlands; ^3^ Laboratory of Molecular Biology, NIDDK, NIH, Bethesda, MD, USA; ^4^ Program in Cellular and Molecular Medicine, Boston Children's Hospital, Boston, MA, USA; ^5^ Department of Stem Cell and Regenerative Biology, Harvard University, Cambridge, MA, USA; ^6^ Harvard Stem Cell Institute, Harvard University, Cambridge, MA, USA; ^7^ Division of Transfusion Medicine, Department of Laboratory Medicine, Boston Children's hospital, Harvard Medical School, Boston, MA, USA

**Keywords:** recombination activation gene 1, RAG1, genome editing, immunodeficiency, CRISPR/Cas9

## Abstract

Mutations in the Recombination Activating Gene 1 (*RAG1*) can cause a wide variety of clinical and immunological phenotypes in humans, ranging from absence of T and B lymphocytes to occurrence of autoimmune manifestations associated with expansion of oligoclonal T cells and production of autoantibodies. Although the mechanisms underlying this phenotypic heterogeneity remain poorly understood, some genotype-phenotype correlations can be made. Currently, mouse models of Rag deficiency are restricted to *RAG1*^−/−^ mice and to knock-in models carrying severe missense mutations. The Clustered Regularly Interspaced Short Palindromic Repeat (CRISPR)/Cas9 system is a novel and powerful gene-editing strategy that permits targeted introduction of DNA double strand breaks with high efficiency through simultaneous delivery of the Cas9 endonuclease and a guide RNA (gRNA). Here, we report on CRISPR-based, single-step generation and characterization of mutant mouse models in which gene editing was attempted around residue 838 of RAG1, a region whose functional role had not been studied previously.

## INTRODUCTION

The *Recombination Activating Gene 1* (*RAG1*) and *RAG2* are critical for T and B cell development. The RAG1 and RAG2 proteins form a complex that introduces DNA double strand breaks (DSBs) at the recombination signal sequences (RSSs) that flank the Variable (V), Diversity (D) and Joining (J) gene segments of the immunoglobulin (Ig) and T-cell receptor (TCR) genes, thereby initiating the process of V(D)J recombination that permits expression of Ig and TCR molecules [1]. Mutations in *RAG1* or *RAG2* can lead to a wide variety of clinical and immunological phenotypes in humans, including complete absence of T and B cells (T- B- Severe Combined Immunodeficiency (SCID)) [2]; Omenn syndrome (OS) with lymphadenopathy, increased serum IgE, eosinophilia, erythroderma and detectable autologous, oligoclonal and activated T lymphocytes [3-9]; RAG deficiency with expansion of TCRγδ^+^ T cells [9]; atypical/leaky SCID (LS) with some T and B cells but no typical OS features [10, 11]; Combined Immunodeficiency with granuloma and/or autoimmunity (CID/G/A) [12-14], and CD4 lymphopenia [15]. The mechanisms underlying such phenotypic heterogeneity remain poorly defined, but in the past years some genotype-phenotype correlation has emerged [16, 17].

**Figure 1:**
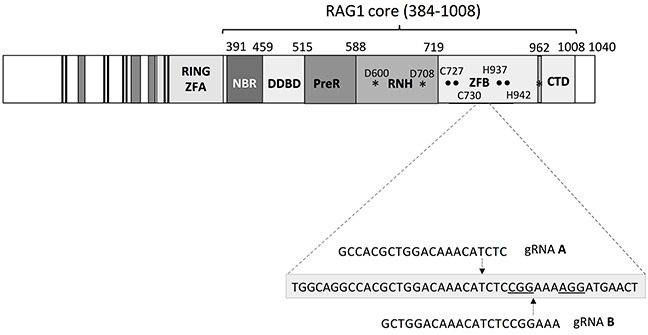
RAG1 structure and gRNA design Two gRNAs (gRNA A and gRNA B) were designed to target the region around residue 838. Here the protospacer region of each gRNA is shown. PAM sequence (NGG) is underlined. Zinc Finger A (ZFA) and Zinc Finger B (ZFB), Nonamer binding region (NBR) and DNA dimerization and binding domain (DDBD), pre-RNase (preR), the catalytic RNase H-like (RNH) domain and C-terminal domain (CTD). Residue numbers are given for the boundaries of the different domains. Catalytic residues D600, D708 and E962 are denoted with an asterisk (*). ZFB (one domain) consists of two binding regions (residues 727/730 and residues 937/942), denoted by (•). The region in between the two zinc binding regions (that form one domain) was targeted.

The RAG1 protein is highly conserved between humans and mice. Mouse RAG1 consists of a RING finger (ZFA), a nonamer binding region (NBR), a dimerization and DNA-binding domain (DDBD), an RNase H-like catalytic domain containing the metal-chelating carboxylates D600, D708 and E962, and a large insertion between residues D708 and E962, which includes two Zinc binding regions (one is formed by C727 and C730, the other by H937 and H942) that together form one zinc finger binding domain (ZFB) [18] (Figure 1). The stretch in between these residues is of unknown function, as also shown by two recent structural studies [17, 18]. The C-terminal domain (CTD) starts immediately after the catalytic residue E962 and interacts extensively with the DDBD domain. Based on recent crystallography data, mutations causing SCID and OS can be grouped in four classes. The first class of mutations destabilizes the tertiary structure, as is the case for mutations involving the zinc binding sites. The second class of mutations involves domains important for DNA binding. The third class of mutations involves the catalytic RNase H-like domain. Lastly, the fourth class involves the RAG1/RAG2 interface [18].

In addition to the original knock-out models, *Rag1*^−/−^ [19] and *Rag2^−/−^* [20], characterized by complete absence of T and B cells, three mouse knock-in models of OS and LS have been reported: the hypomorphic *Rag2* mutation R229Q [21] (involving the RAG1/RAG2 interface), the hypomorphic *Rag1* mutation S723C [22] (close to one of the zinc binding regions) and the R972Q [23] mutation (affecting the CTD). However, missense mutations in regions other than the NBR, DDBD, catalytic domain or zinc binding domain often show higher residual V(D)J recombination activity and are frequently seen in patients with less severe and delayed-onset disease, often associated with autoimmunity, as was the case for the human mutation R841W (mouse R838W) [16]. Therefore, we decided to target the region around residue 838 of the RAG1 locus, which falls within the catalytic residues 708 and 962 and does not involve any zinc binding regions.

Traditionally, in order to generate mouse models of human diseases, gene targeted embryonic stem cells (ESCs) are electroporated with a DNA template containing the desired mutation in the gene of interest flanked by homology arms. Usually, an excisable antibiotic resistance gene is also introduced in one of the homology arms to facilitate identification and selection of targeted clones. Homology-directed repair (HDR) is a low efficiency process that permits to replace the endogenous target ESC genomic sequence with that provided by the DNA template. Upon *in-vitro* culture under antibiotic pressure and screening, by polymerase chain reaction (PCR), ESC clones that have been successfully targeted with the desired sequence are initially selected and expanded, and are then injected into blastocysts, and implanted in pseudo-gestating females. The resulting chimeric offspring animals have to be further bred until the introduced mutation is transmitted through the germline. Overall, this is a lengthy, rather inefficient, and expensive process.

Recently, the Clustered Regularly Interspaced Short Palindromic Repeats (CRISPR)/CRISPR associated 9 (Cas9) system has emerged as a novel and powerful gene editing platform. The Cas9 nuclease is directed to a specific DNA site by a guide RNA (gRNA), allowing targeted introduction of DNA double strand breaks (DSBs), that can be repaired through the error-prone non-homologous end-joining (NHEJ) machinery, which often introduces insertions and/or deletions (indels) at the DNA DSB site, leading to frameshift and early termination and hence gene disruption. Alternatively, if a homologous DNA template sequence with a desired mutation is provided along with Cas9 and the gRNA, homology-mediated repair permits replacement of the endogenous target DNA sequence with the mutated sequence of interest. Cas9-based gene editing has therefore emerged as a novel and powerful strategy to generate knock-out (KO) and knock-in (KI) mouse models. Importantly, Cas9 and gRNA with or without a homologous single strand DNA oligonucleotide (ssODN) can be directly injected into multiple mouse zygotes that are then implanted into foster mothers, with no need for further selection. This method has significantly improved the efficiency of generating genetically modified mouse models, with significant containment of costs and time [24-26].

Here we describe multiple *Rag1* mouse models generated with high efficiency in a single step by targeting zygotes with Cas9 and a *Rag1*-specific gRNA. Several mice with various mutations at and around residue 838 were studied, providing insights into the functional role of the region. This study also offers proof of principle that a similar strategy can be applied to develop novel knock-in mouse models that may help interrogate the specific role of individual amino acid residues within this region.

## MATERIALS AND METHODS

### Design of Rag1-specific gRNAs

In order to target the region around residue 838 of RAG1, a series of gRNAs were designed. In order to select gRNA protospacer sequences with limited off-site targeting, NCBI blast was used to search the mouse genome for sequence similarity. gRNA protospacer sequences were selected that had no perfect matches, 1-base mismatches, or 2-base mismatches, and as few 3-base mismatches as possible, in other regions of the mouse genome than the target sequence. As a result of this process, two different gRNAs (gRNA A and B) were selected which would introduce a DSB between nucleotide c. 2508 and c. 2509 (gRNA-A) and between nucleotide c. 2514 and c. 2515 (gRNA-B), respectively (Figure 1). Each protospacer region was cloned in a gRNA cloning plasmid (Addgene) [27].

### Testing of the efficiency of gRNA-mediated introduction of targeted DSBs

The efficiency of gRNA-A and gRNA-B in mediating targeting of the *Rag1* gene was compared in murine ESCs. ESCs were grown cultured in media consisting of DMEM high glucose supplemented with 15% FCS, 1% Pen/strep, 1% L-glutamine, 1% Non-essential amino acids and 10^^−4^ M Beta mercapto-ethanol and 10^3^U/ml LIF. Cells were transfected with 2.5μg of the pCas9-GFP plasmid (Addgene) and either 2.5μg gRNA-A or 2.5μg gRNA-B using the 4D Amaxa nucleofector (Lonza), using kit P3 and the protocol provided by the manufacturer. Forty-eight hours post-nucleofection, GFP-positive ESCs expressing Cas9-GFP were selected by FACS sorting. Genomic DNA was isolated and subjected to high throughput sequencing (HTS) using barcoded primers and the 454 GS Junior apparatus (Roche). For each gRNA, the efficiency of on-site targeting was defined as the percentage of reads containing an indel spanning the target site.

### Zygote injection and analysis of Rag1 gene targeting in the offspring

Two hundred and fifty C57BL/6 zygotes were injected with 100 ng/μl Cas9 mRNA (purchased from System Biosciences Cat#CAS500A-1) and 50 ng/μl gRNA A (generated from PCR product using Megashortscript T7 kit Life Technologies) into the cytoplasm, as previously described [24]. A PCR of the gRNA was performed to add a T7 promoter to the gRNA sequence as previously described [25]. Two hundred and thirty live embryos were implanted into seven recipient foster mothers. Pups were born two and a half weeks later. Tail gDNA of three-week-old pups was analyzed by PCR and Sanger sequencing for the presence of mutations in the *Rag1* gene. To this purpose, the genomic region spanning 2290 - 2723 (433 bp) was amplified using the following primers:

Forward primer: 5′-CGTATCATGAGTCCGTGGAA-3′

Reverse primer: 5′-TGACAGAGGGACTCTGACAC-3′

PCR was performed using a 20μl reaction with Phusion Hot start II (Lifetechnologies Cat# F-549L). The manufacturer's PCR settings were followed, using GC buffer 5X, a final concentration of 3% DMSO and an annealing temperature of 60C. For sixteen mice, in which no amplification products were obtained with the primers reported above, a PCR reaction was performed with primers that span a 1500 bp region of *Rag*1 around the Cas9-induced cleavage site:

Forward primer: 5′-TCTGGACTTGCCTCCTCTGT-3′

Reverse primer: 5′-CCATTGAATCTTGGCTTTCC-3′

### Breeding and control mice

The five mice with in-frame deletions (ID 25, 37, 48, 49, 51) and one with a frameshift deletion (ID 41), collectively referred to as Δ*Rag1* mice, were sacrificed and analyzed without breeding (Table 1). To segregate the three alleles, female mouse 31 was bred with a wild type (*wt*) C57BL/6 male resulting in offspring with the different mutations. F1 mice with heterozygous H836Q mutation (*Rag1*^+/H836Q^ mice) were bred to get F2 mice homozygous for the H836Q mutation (*Rag1 H836*Q mice). *Rag1*^−/−^ mice were purchased from the Jackson Laboratory. All mice described in this manuscript were on a C57BL/6 background.

**Table 1:** Genotype results

mouse ID	Nucleotide	Aminoacid
14	c. [2105_3014del]	p. [Phe702Phefs*4]
30	~700 bp del	
35	~600 bp del	
10	c. [2503_3021del] c. [c.2508_2999del]	p. [Lys835_Asn1007] p. [His836Glnfs*41]
50	c. [2505_2920del]	p. [Lys835Asnfs*9]
2	c. [2500_2811del]	p. [Asp834_His937del]
18	c. [2497_2785del]	p.[Leu833Argfs*45]
34	c.[2501_2763del]	p.[Asp834Aspfs*5]
40	c.[2508_2739del]	p. [His836Hisfs*61]
17	c.[2503_2671del]	p.[Lys835Profs*112
23	c. [2507_2671del]	p. [His836Profs*150
8	c.[2363_2514del]; c.[2502_2524del]	p.[Val788Glufs*29]; p.[Asp834Glufs*26]
21	c. [2494_2642del]	p. [Thr832Alafs*18]
4	c.[2503_265del]	p.[Lys835Profs*15]
25*	c. [2508_2627del]	p. [Leu837_His876del]
36	c. [2507_2516del]; c. [2507_2585del]	p. [His836Glnfs*2]; p.[His836Glnfs*2]
42	c. [2464_2512del]; c. [2508del]	p. [Ser822Glyfs*3]; p. [His836Hisfs*7]
31**	c. [2508T>A]; c. [2462_2509del]; c. [2508del]	p. [His836Gln]; p. [Ala821Valfs*5]; p. [His836Hisfs*6]
20	c. [2509_2540del]	p. [Leu837Glufs*20]
44	c.[2501_2515delinsCGCTTTTGGTAGATGCCTTCCCATCCACTCGGTT]	p. [Asp834Alafs*12]
37*	c. [2507_2533del]; c. [2510del]	p. [His836_Lys844del]; p. [Leu837Profs*4]
28	c.[2500_2510del]	p.[Leu833Leufsfs*31]
43	c. [2508_2512delinsAAA]	p. [His836Glnfs*31]
33	c. [2503_2516del]	p. [Lys835Lysfs*29]
9	c. [2503_2516del]	p. [Lys835Lysfs*29]
16	c. [2503_2516del]	p. [Lys835Lysfs*29]
27	c.[2472_2505delinsAAAGTT;2508_2509insTC]; c. [2503_2516del]	p. [Glu824Glufs*35]; p. [Lys835Lysfs*29]
29	c. [2502_2515del]; c. [2509_2510delinsGTCCTTT]	p. [Asp834Glufs*29]; p. [Leu837Valfs*6]
39	c. [2502_2515del]; c. [2508insC]	p. [Asp834Glufs*29]; p. [His836Hisfs*32]
52	c. [2502_2515del]	p. [Asp834Glufs*29]
13	c. [2508delinsAA] c. [2502_2515del]	p. [His836Glnfs*32] p. [Asp834Glufs*29]
5	c. [2502_2515del]	p. [Asp834Glufs*29]
22	c. [2502_2515del]	p. [Asp834Glufs*29]
38	c.[2499_2512del]	p.[Leu833Leufs*30]
45	c.[2499_2512del]; c. [2508_2509del]	p.[Leu833Leufs*30]; p. [His836Hisfs*31]
41*	c. [2496_2509del]	p. [Thr832Thrfs*31]
3	c. [2505_2508del]; c. [2507_2556del]	p. [Lys835Asnfs*5]; p. [His836Leufs*15]
32	c. [2506_2508delinsTG]	p. [His836Cysfs*5]
48*	c. [2493_2495del; 2512C>T]; c. [2493_2495del]	p. [Thr832del; Trp838del]; p. [Thr832del]
49*	c. [2507_25093del]; c. [2505-2573delinsG]	p. [His836del]; p. [His846Aspfs*9]
51*	c. [2504_2509del]; c. [2505del; 2508_2509del]	p. [Lys835Ile; His836_Leu837del]; p. [Lys835_Leu837delinsAsn835_Ile836]
6	c. [2509_2510del]	p. [Leu837Profs*30]
19	c. [2507_2508del]	p. [His836Profs*31]
47	c. [2507_2508del]	p. [His836Profs*31]
53	c. [2508_2509del]	p. [His836Hisfs*31]
26	c. [2509_2510del] c. [2508_2509insT]	p. [Leu837Profs*30]; p. [Leu837Serfs*31]
15	c. [2508_2509insA]	p. [Leu837Thrfs*31]

When for each allele a different sequence is given (heterozygous), two different sequences were seen by Sanger sequencing. When only one sequence is given, this means only one sequence was amplified and detected by Sanger sequencing. This could either be due to an identical indel on both alleles (homozygous) or the second allele was not amplified by PCR due to a large deletion or translocation.

* 5 mice with in-frame deletions and one with frameshift (as one example), were further analyzed by FACS without further breeding.

** Mouse 31 showed 3 sequences, due to mosaicism and was further bred to separate the alleles.

### FACS analysis of spleen, thymus and bone marrow cells

Total cellularity of thymus, spleen, bone marrow and the distribution of the various T and B cell subsets were analyzed in 8-12 week-old Δ*Rag1* mice and *Rag1 H836Q* mice. Both groups were compared to age-matched wild type (*wt*) and *Rag1 ^−/−^* mice (Jackson Laboratory) [19]. Splenocytes were stained with a T cell panel consisting of CD4-PB (Ebioscience), CD8-PE/Cy5 (BD), CD3 PE-Cy7 (Biolegend) and a B cell panel consisting of B220-APC (Biolegend), IgM-PE-cy5 (Ebioscience), CD43-PE (Ebioscience). Bone marrow cells were stained with B220-APC (Biolegend), IgM-Pe-Cy5 (Ebioscience), and CD43-PE (Ebioscience) antibodies. Thymocytes were stained with CD3-PE-Cy7 (Biolegend), CD4-PB (Ebioscience), CD8-PE-Cy5 (BD), CD44-FITC (BD), and CD25-PE (Ebioscience) antibodies, upon excluding B220 (Biolegend), Ter119 (Biolegend) and MAC1 (Biolegend) positive cells. FACS gating strategies are shown in Supplementary Figures S1 to S4 (S1-S4). Standard FSC/SSC live gates and FSC/SSC lymphocyte gates were used.

### Statistical analysis

Two-tailed unpaired *t*-test was used to compare results in Δ*Rag1* vs. *wt* mice and to compare *Rag1 H836Q* to *wt* mice.

## RESULTS

In order to compare the ability of gRNA-A and gRNA-B to induce Cas9-mediated introduction of on-site DSBs at the *Rag1* locus, we analyzed the frequency of indels as detected by high-throughput sequencing (HTS) in nucleofected ESCs. Any deletion and/or insertion spanning the cutting site was counted. A higher frequency of on-site targeting was detected with gRNA-A (57% vs 15%). Therefore, gRNA-A was selected for injection into the murine zygotes.

Upon injection of Cas9 mRNA and gRNA-A into 250 C57BL/6 zygotes, 230 live embryos were obtained and transferred into seven female recipient mice. After 18-20 days, 53 live pups were born. At 3 weeks of life, Sanger sequencing of the *Rag1* gene was performed using the tail gDNA as a template. Results of genotyping are shown in Table 1. For sixteen mice (ID 1, 2, 7, 10, 11, 12, 14, 18, 21, 24, 30, 34, 35, 40, 46 and 50), the initial amplification of 433 bp around the DNA cleavage site did not yield any product. Upon performing PCR with primers that span a 1.5 kb region around the cleavage site, smaller fragments were detected in ten of these mice (ID 2, 10, 14, 18, 21, 30, 34, 35, 40 and 50), indicating the occurrence of intragenic deletions, whose boundaries were defined by Sanger sequencing (Table 1). For six mice (ID 1, 7, 11, 12, 24, 46) no products were obtained even with this strategy, suggesting occurrence of either an even larger deletion or chromosomal translocation [28]. These six mice with undefined mutations were omitted from any further analysis. In all other cases, Sanger sequencing showed either two distinct sequences, corresponding to different indels on different alleles, or one single sequence with an indel spanning the cutting site. The latter may reflect either homozygosity for that indel, or compound heterozygosity with the second allele failing to amplify due to a large deletion or translocation.

In summary, all 53 mice showed bi-allelic targeting of the *Rag1* locus. Forty-two of them showed deletions, 1 showed only an insertion, 9 showed a combination of insertions and deletions, and one mouse (ID 31, indicated by two asterisks in Table 1) showed somatic chimerism, with three distinct alleles. Upon subcloning, it was established that these mutant alleles corresponded to a c.2508T>A nucleotide substitution (p.His836Gln) a c. 2462_2509del (p. Ala821Valfs*5), and a 2508del (p.His836Hisfs*6) (Table 1). Five mice (ID 25, 37, 48, 49, 51; indicated by an asterisk in Table 1) showed in-frame deletions or insertions on one allele, and either frameshift deletions or large deletions on the other allele. The sizes of the in-frame deletions corresponded to one (residue 836 in mouse 49), two (residue 832 and 838 in mouse 48), nine (residue 836-844 in mouse 37) or forty amino acids (residue 837-876 in mouse 25) (Table 1). For one mouse (ID 51), a 6 bp deletion on one allele resulted in substitution of Lysine to Isoleucine at position 835 (Lys835Ile) and deletion of residues 836 and 837 (p. Lys835Ile; His836_Leu837del). On the other allele, a 3 bp deletion resulted in deletion of residue 837 with a substitution of Lysine to Asparagine in position 835 and substitution of Histidine to Isoleucine in position 836 (p. Lys835_Leu837delinsAsn835_Ile836).

**Figure 2:**
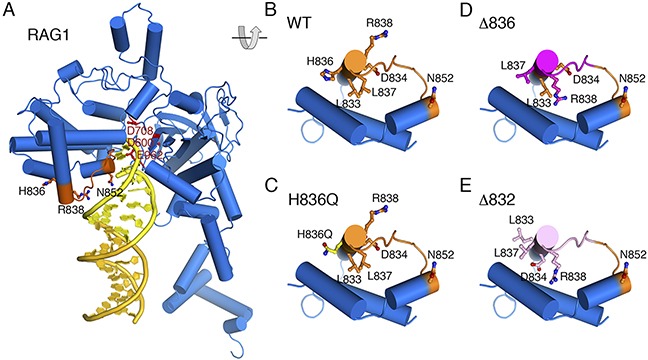
Structural model of RAG1-RSS complex **A.** Wild type RAG1. RAG1 core is shown in blue. The yellow portion of the DNA is 7bp (heptamer), and the orange colored DNA represents the spacer region. The DNA contact region (residues 838-852) is highlighted in brown. **B.** wild type RAG1 buta zoom in view of the protein helices surrounding the DNA binding region and rotated ~90° indicated by the grey diagram. **C.** The H836Q mutation (Histidine substituted by Glutamine). Glutamine has the hydrogen bond donor (NH2 group, shown in blue) and acceptor (carboxyl oxygen, shown in red) at positions equivalent to those in the imidazole group of Histidine (one ring NH as donor and the other ring N as acceptor). **D.** The possible consequence of deletion of H836 in the middle of an alpha helix. The residues after H836 all shift by one register and L837 (Leucine) would occupy the H836 position, meaning a hydrophobic residue in a hydrophilic environment. R838 (Arginine) shifts into a hydrophobic pocket occupied by L837 in WT RAG1, thus destabilizing the protein structure and interfering with all RAG1 functions from DNA binding to cleavage. **E.** The predicted effect of deleting residue 832 is similar to deleting residue 836, which is shifting the register of an alpha helix by one residue and resulting in charged residues in the hydrophobic core. Since this deletion occurs one helical turn before H836, the effect is even more severe. D834 is placed in the position of L833 in addition to R838 being placed in the L837 position.

The human RAG1 H839Q mutation, corresponding to H836Q in, has not been reported in patients, nor in public databases of genomic variants in the general population (dbSNP, 1000 genomes, ExAC). The human H839Q variant was however predicted to be damaging by SIFT, probably pathogenic by Polyphen-2, but neutral by Snap and SNP&Go program analyses. To predict the structural consequences of this mutation, a model was developed based on crystal structure of mouse RAG1 [18]. Mouse RAG1 residues R838 - N852 are predicted to have extended interactions with the heptameric region of RSS DNA (Figure 2A and 2B). Therefore, any deletion in this region is predicted to have a deleterious effect on RSS binding. Residue 836 in particular is important for interaction between two alpha helices in RAG1. However, Histidine and Glutamine have similar hydrogen bonding potential. Glutamine has the hydrogen bond donor (NH2 group) and acceptor (carboxyl oxygen) sites at positions equivalent to those in the imidazole group of Histidine (one ring NH as donor and the other N as acceptor). Therefore, substituting Histidine with Glutamine should have little effect on protein interactions and none on DNA binding (Figure 2C). Altogether, structural modeling predicted the H836Q mutation in mouse RAG1 to have little or no effect on protein expression and function. In contrast, Figure 2D shows the possible consequence of deletion of H836 (observed in mouse 49) within an alpha helix. Assuming the alpha helix remains stable, the residues after H836 all shift by one register, and L837 thus would take the position of H836 in the deletion mutant, which would place a hydrophobic residue in a hydrophilic environment. Moreover, the R838 (Arginine) is shifted into a hydrophobic pocket normally occupied by L837 (Leucine) in wild type RAG1. Arginine is a longer amino acid than Leucine and has a more positive charge. Therefore, deletion of H836 is predicted to wreck the protein structure and interfere with RAG1 functions ranging from DNA binding to cleavage. The effect of deleting T832 (observed in mouse 48) is similar to deletion of 836, shifting the register of this alpha helix by one residue and resulting in charged residues in the hydrophobic core. Since T832 is an earlier residue in the same helix as H836, deleting T832 would be expected to have an even more severe effect, placing D834 in the position of L833, in addition to R838 being placed in the former position of L837 (Figure 2E).

Next, we assessed the functional consequences of the various in-frame deletions or insertions to T and B cell development, in the gene-targeted mice. Since bi-allelic out-of-frame deletions in this region uniformly result in a complete T- B- SCID phenotype in humans, we focused our attention on mice carrying in-frame deletions or insertions. For mouse 31, which was found to be a somatic chimera, we segregated the 3 distinct mutant alleles by sequential breeding with *wt* mice. Intercrossing of heterozygous *Rag1*^+/H836Q^ mice yielded mice that were homozygous for the p.H836Q mutation, which were kept for further analyses. Finally, one mouse (ID 41), with a frameshift deletion leading to a stop codon at position 863, was included as a positive control of severe *Rag1* locus disruption.

Peripheral blood of the five RAG1 targeted mouse strains with in-frame deletions and of the mouse with the frameshift mutation (collectively referred to as Δ*Rag1* mice), all showed complete absence of peripheral T and B cells by FACS analysis (Supplementary Figure S1).

**Figure 3:**
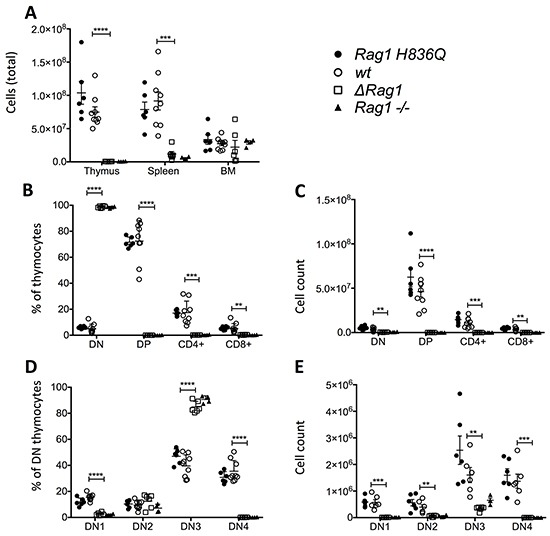
Total Cell counts and FACS Thymus **A.** Total cell counts in Thymus, bone marrow (BM) and spleen. **B.** FACS ofThymocyte subsets (%) (excluding B220+, Ter119+ MAC1+). **C.** Absolute cell counts of 3.B. **D.** Subsets of DN (%). **E.** Absolute cell counts of 3.D. *Rag1 H836Q* (n=6), *wt* (n=9), *Rag1* knock-out (*Rag1−/−)* (n=4). *ΔRag1* (n=6) are mouse 25, 37, 41, 48, 49 and 51 with each different indels (Table 1). Two tailed, unpaired T-tests between *ΔRag1* and *wt* ** P ≤ 0.01, *** P ≤ 0.001, **** P ≤ 0.0001. There was no significant difference between *Rag1 H836Q* and *wt.*

Total thymic cellularity in Δ*Rag1* mice was markedly decreased compared to that in *wt* mice, and consistent with that in *Rag1^−/−^* mice (Figure 3A). Immunophenotypic analysis of thymocyte subsets in these mice showed an arrest at the CD4^−^ CD8^−^ double negative stage (Figure 3B and 3C), and in particular at the DN3 (CD44^−^ CD25^+^) stage of differentiation (Figure 3D and 3E), which marks initiation of *Rag* gene expression. Importantly, a similar severe phenotype was observed also in mouse 49 with deletion of three nucleotides corresponding to deletion of H836 (Table 1). By contrast, thymic cellularity and thymocyte subset distribution were comparable in mice homozygous for the H836Q mutation (*Rag1 H836Q)* and in *wt* mice.

**Figure 4:**
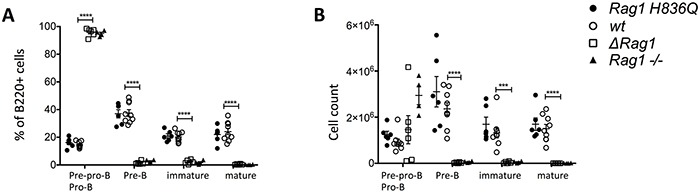
FACS Bone Marrow **A.** Subsets of B220+ cells (%). Pre-pro and pro-B cell= B220^+^ CD43^+^ IgM^−^, Pre-B cells = B220^+^ CD43^−^ IgM^−^, immature B cells = B220^low^ CD43^−^ IgM^+^, mature B cells= B220^hi^ CD43^−^ IgM^+^. **B.** absolute cell counts of 4.A. *Rag1 H836Q* (n=6), *wt (*n=9), *Rag1* knock-out (*Rag1−/−)* (n=4). *ΔRag1* (n=6) are mouse 25, 37, 41, 48, 49 and 51, each with different indels (Table 1). Two tailed, unpaired T-tests between *ΔRag1* and *wt*. *** P ≤ 0.001, **** P ≤ 0.0001. There was no significant difference between *Rag1 H836Q* and *wt.*

In the bone marrow the Δ*Rag1* showed a complete lack of pre-B cells (B220^+^ CD43^−^ IgM^−^), immature B cells (B220^low^ CD43^−^ IgM^+^) and mature B cells (B220^hi^ CD43^−^ IgM^+^) and an arrest at pre-pro and pro-B cell (B220^+^ CD43^+^ IgM^−^) stage of B cell differentiation (Figure 4A and 4B). A similar pattern was observed in *Rag1^−/−^* mice. In contrast, B cell counts and cell subset distribution were comparable in *Rag1 H836Q* and *wt* mice.

**Figure 5:**
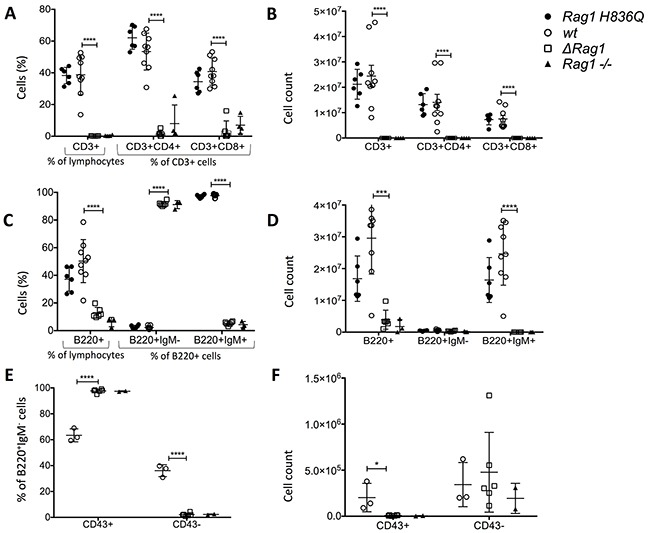
FACS Spleen **A.** CD3+ % of lymphocytes (SSC, FSC gate) and CD4+ and CD8+ as % of CD3+ cells. **B.** CD3, CD4, CD8 absolute cell counts of 5.A. **C.** B220+ % of lymphocytes (SSC, FSC gate) and precursos and immature (B220^+^IgM^−^) vs mature B cells (B220^+^IgM^+^) (% of B220+ cells). **D.** absolute cell counts of 5.C. **E.** Gated on B220^+^IgM^−^ cells to distinguish pre-B (CD43-) cells from pro-B cells (CD43+). **F.** Absolute cell counts of 5.E. *ΔRag1* compared to *wt* and *Rag1−/−.* Two tailed, unpaired T-tests between *ΔRag1* and *wt*. * P ≤ 0.05, **** P ≤ 0.001, **** P ≤ 0.0001. No significant difference between *Rag1 H836Q* and *wt* (5.A-D, not done for 5.E and 5.F).

In the spleen, Δ*Rag1* mice showed marked reduction of total cellularity, associated with lack of CD4^+^ and CD8^+^ cells (Figure 5A and 5B). Furthermore, only B220^+^ IgM^−^ B cells, but no mature B220^+^ IgM^+^ B cells were detected (Figure 5C and 5D). The IgM^−^ B cells were all CD43^+^, corresponding to pro-B cells, whereas in *wt* mice a proportion of the IgM^−^ B cells were CD43^−^, representing pre-B cells (Figure 5E and 5F).

**Figure 6:**
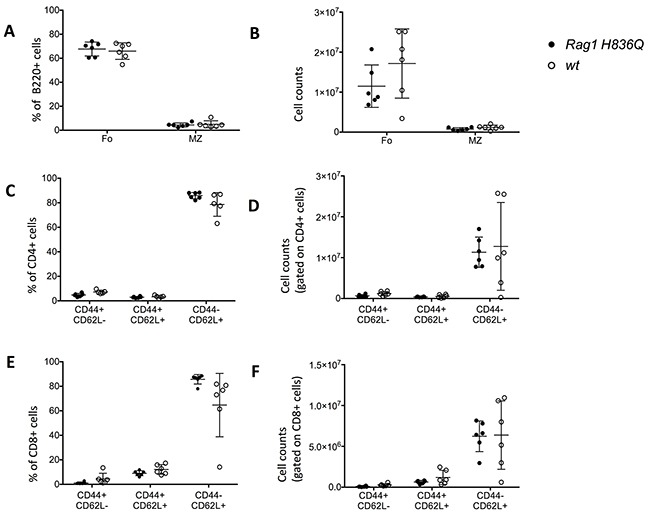
FACS Spleen **A.** Follicular (Fo) (B220^+^, CD93^−^, CD23^+^CD21^−^), and marginal zone (MZ) (B220^+^, CD93^−^, CD23^−^CD21^+^) B cells in the spleen (% of B cells). **B.** Absolute cell counts of 6.A. **C.** Activated (CD44^+^CD62L^−^), memory (CD44^+^CD62L^+^) and naïve (CD4^−^CD62L^+^) CD4+ T cell subsets in the spleen (% of CD4+). **D.** Absolute cell counts of 6.C. **E.** Activated (CD44^+^CD62L^−^), memory (CD44^+^CD62L^+^) and naïve (CD4^−^CD62L^+^) CD8+ T cell subsets in the spleen (% of CD8+). **F.** Absolute cell counts of 6.E. Two tailed, unpaired T-tests between *Rag1 H836Q* (n=6) and *wt* (n=6). No significant difference between *Rag1 H836Q* and *wt.*

By contrast, the percentages and absolute counts of CD4^+^ and CD8^+^ T cells were comparable in *Rag1 H836Q* and *wt* mice (Figure 5A and 5B), and so were the percentages and absolute counts of mature B220^+^ IgM^+^ B cells (Figure 5C and 5D), marginal zone B cells (MZ) and follicular B cells (Fo) (Figure 6A and 6B). Finally, CD4^+^ and CD8^+^ cells had a normal distribution of activated (CD44^+^CD62L^−^), memory (CD44^+^CD62L^+^) and naïve (CD4^−^CD62L^+^) cells in *Rag1 H836Q* mice compared to *wt* mice (Figure 6C-6F).

## DISCUSSION

CRISPR/Cas9 is a highly efficient and versatile tool for performing genome-editing in mammalian cells *in-vitro* and for generating animal models for *in-vivo* studies. Here we showed that CRISPR/Cas9 can be efficiently used to generate several unique *Rag1* murine models in six weeks counting from the start of zygote injection to an F0 generation of weaning age, circumventing months of breeding as required for the traditional ES cell blastocyst injection. In addition, no complicated cloning of constructs is required, and several models can be obtained at once. We showed that when testing different gRNAs in *vitro* and selecting the gRNA with the highest DNA cleavage efficiency, zygote injection can lead to a targeting efficiency of 100%, as was seen previously for other genes using the same injection conditions [24]. Using this strategy, we were able to evaluate the effects of in-frame deletions in residues 832-877 of RAG1, a region of unclear function that does not contain any catalytic residues nor is involved in zinc binding. The mice we studied had deletions of one (residue 836 in mouse 49 and residue 832 in mouse 48), two (residue 832 and 838 in mouse 48), nine (residue 836-844 in mouse 37) or forty amino acids (residue 837-876 in mouse 25) (Table 1). All of these in-frame deletions led to a severe phenotype, with a B cell development block at the stage of pre-pro and pro-B cells and a T cell development block at DN3.

The use of various algorithms had yielded controversial results with regard to pathogenicity of the H836Q mutation. However, structural modeling studies suggested that this would be a neutral change. Indeed, analysis of the *in-vivo* immunological phenotype of mice with a homozygous H836Q mutation showed intact T and B cell development. Altogether, these data indicate the superiority of structural modeling over in silico predictive pathogenicity tools, and reinforce the importance of *in-vivo* validation to assess the functional effects of missense mutations, especially in this locus that was not tested in vivo in mice.

Although CRISPR/Cas9 has been shown to be a successful and highly efficient genome editing tool for generating *in-vivo* animal models of different species [24, 29-31], somatic mosaicism may arise with this approach, because after pro-nucleus injection, CRISPR/Cas9-mediated cleavage can occur multiple times at various cell number stages in the morula [29], as was observed in mouse 31 in this study. In such cases, sequential breeding is required to segregate the various alleles.

One concern of the CRISPR/Cas9 technique is off-site targeting. We selected gRNAs with as little predicted off-site-targeting activity as possible. At the time of gRNA selection, NCBI blast was used to select the best gRNAs, as described in the Methods section. When novel sequence analysis tools became available (http://crispr.mit.edu/) [32], the predicted off-site targeting activity was also investigated. For gRNA A, there were only 16 possible off-site targets, of which one had 3 mismatches (in position 1, 3 and 19 of the protospacer starting from the PAM site), and all of the others all had at least 4 mismatches, with at least 2 falling in the first 10 bp of the protospacer sequence. It has been reported that mismatches in the first 10 bp of the protospacer are most stringent and make off-site mutagenesis very unlikely[33]. None of the possible off-site targets were in genes known to cause immunodeficiency.

**Figure 7:**
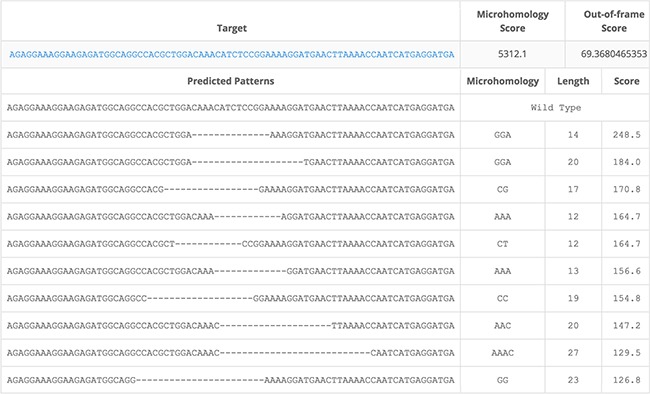
Microhomology prediction Screenshot of output generated by online tool to predict microhomology mediated repair after cleavage by Cas9 with gRNA A. Input: 80bp around cutting site of gRNA A. (http://www.rgenome.net/mich-calculator/).

The different indels that were generated by CRISPR/Cas9 induced cleavage were not completely random. For example, there were 13 mice with 14bp frameshift indels, and only 4 different unique sequences (Table 1). All but one of these sequences showed microhomology of 2 or 3 bp. When using the online tool to predict microhomology for the 80bp around the cutting site, the 14bp sequence that was found in 6 mice, was indeed on top of the list with the highest score for microhomology-mediated repair (http://www.rgenome.net/mich-calculator/) [34] (Figure 7). This is important to realize when selecting a target site and a gRNA.

In summary, we have shown that CRISPR/Cas9 is a highly efficient tool to rapidly generate different mouse models of Rag1 deficiency and to study the functional consequences of such mutations. If a similar strategy is coupled with injection of single-stranded homology DNA template into the zygote, novel knock-in models can be generated rapidly and with high efficiency by means of HDR [24, 26]. This approach would be particularly relevant for modeling the phenotypic heterogeneity that is associated with RAG deficiency in humans by generating multiple mouse models in a short period of time.

## SUPPLEMENTARY FIGURES


