# Supplementary material for: Rapid generation of novel models of RAG1 deficiency by CRISPR/Cas9-induced mutagenesis in murine zygotes
Source: Oncotarget. 2016 Feb 12;7(11):12962–74. doi: 10.18632/oncotarget.7341 (PMC4914335; doi:10.18632/oncotarget.7341)
Supplement: Supplementary file 1 [file oncotarget-07-12962-s001.pdf]

SUPPLEMENTARY FIGURES

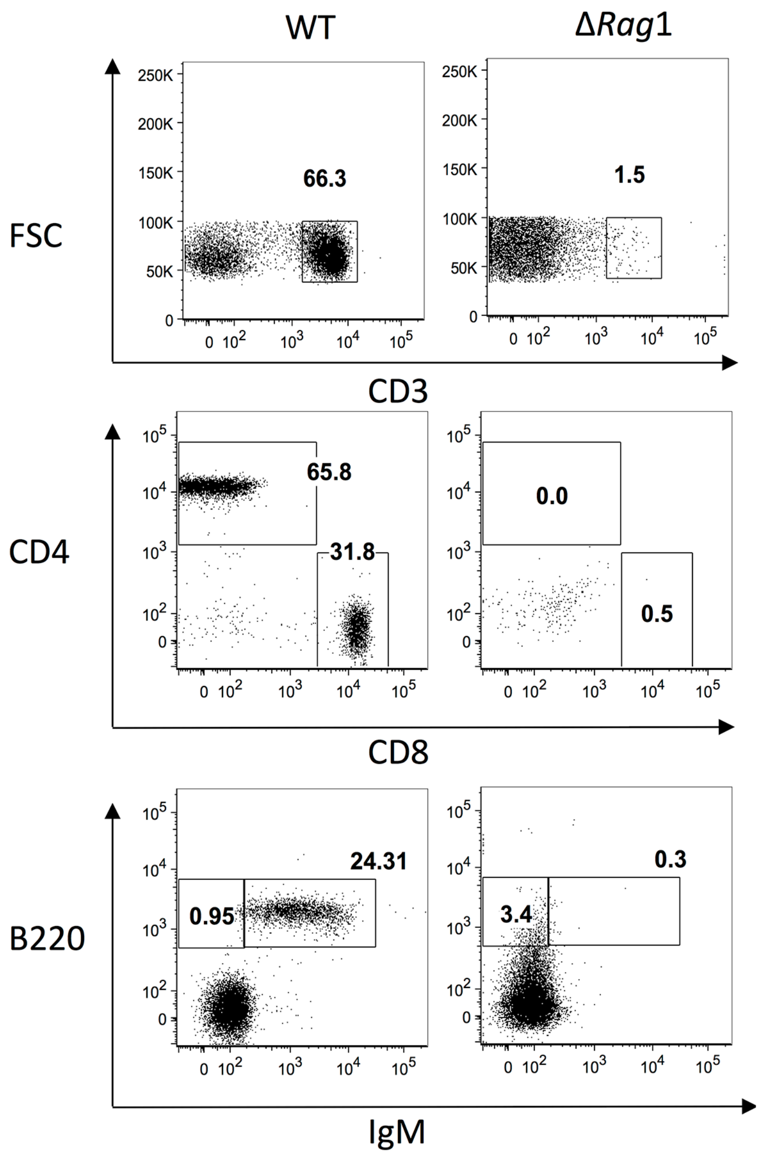

Supplementary Figure S1: FACS peripheral blood. FACS gating strategy to asses T and B cells in peripheral blood of deltaRag1 mice.

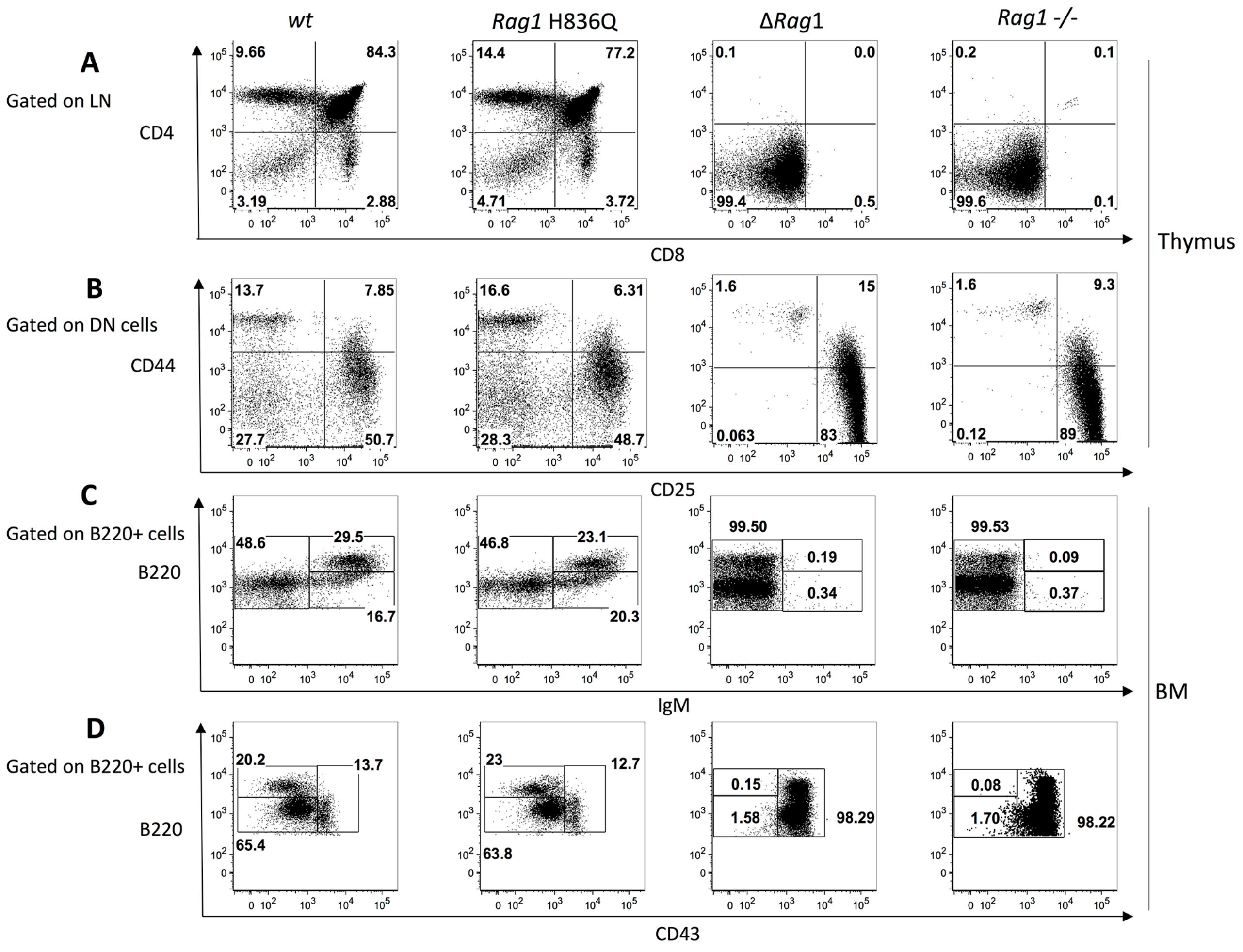

Supplementary Figure S2: FACS Thymus and Bone Marrow. A. FACS gating strategy for thymocyte subsets as shown in Fig.3.B-C. CD4+, CD8+, CD4+ CD8+ (Double Positive (DP)) and CD4- CD8- (Double Negative (DN)). **B.** FACS gating strategy for subsets of DN as shown in Fig. 3.D-E. DN1 (CD44+CD25-), DN2 (CD44+CD25+), DN3 (CD44-CD25+), DN4 (CD44-CD25-). **C.** and **D.** FACS gating strategy for B220+ cells in the bone marrow as described in Fig.4.A-B.

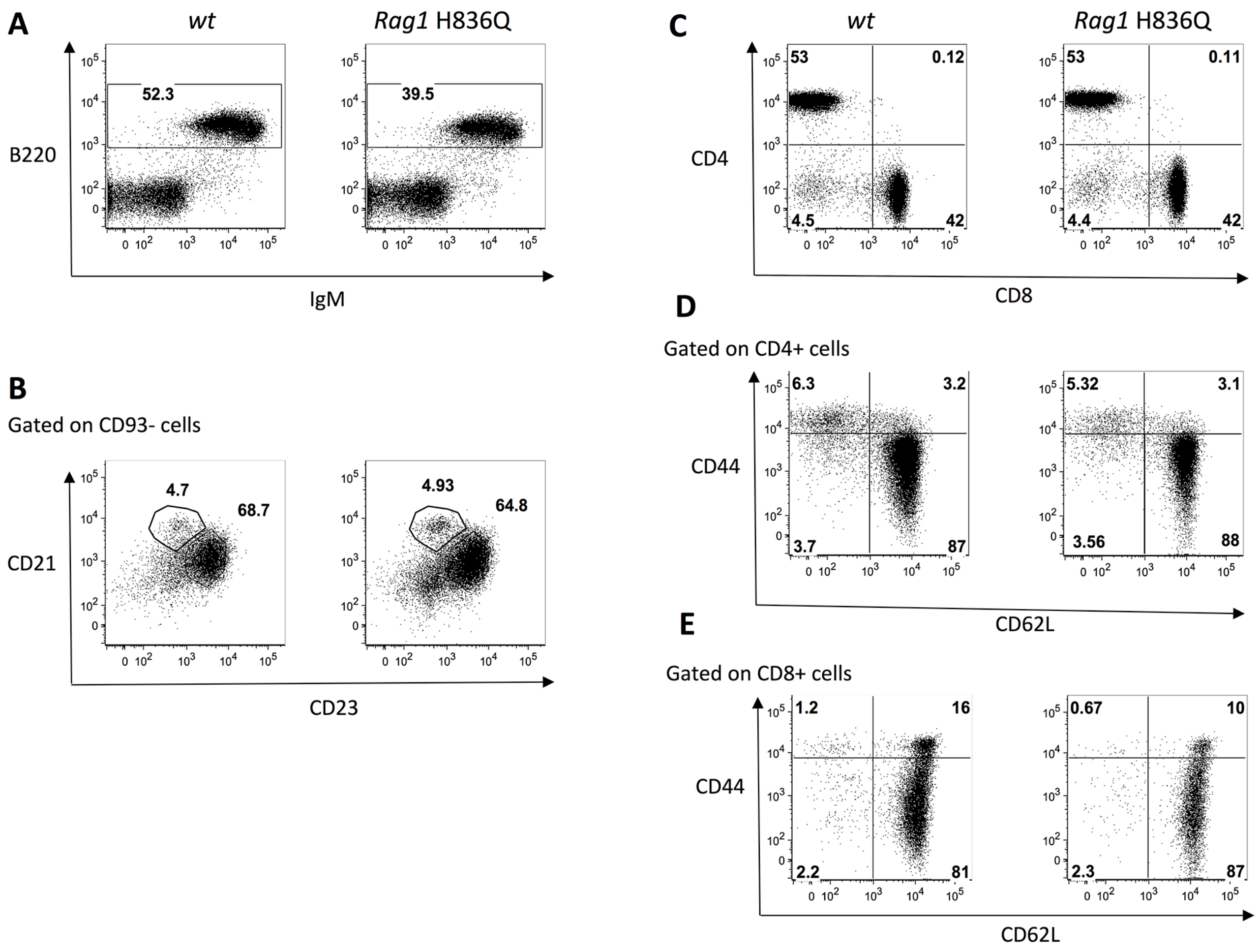

Supplementary Figure S3: FACS Spleen Rag1 H836Q mice. A. FACS gating strategy for Fig.5.C-D. **B.** FACS gating strategy for Fig.6.A-B. **C.** FACS gating strategy for Fig.5.A-B. **D.** FACS gating strategy for Fig.6.C-D. **E.** FACS gating strategy for Fig. 6.E-F.

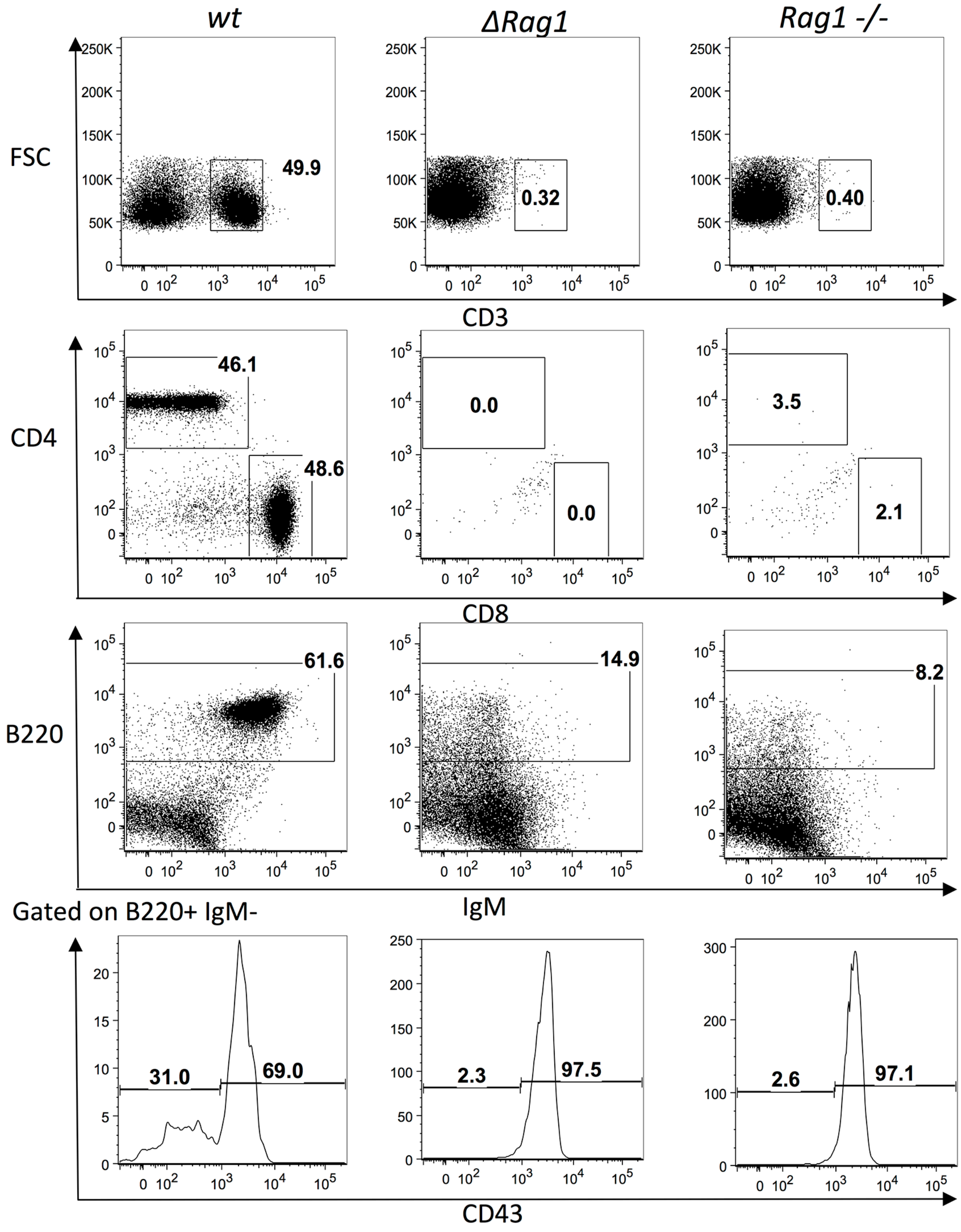

Supplemental Figure S4: FACs spleen ΔRag1mice. FACS gating strategies for Fig.5.
